# Investigating the Relationship Between the Market Orientation Approach of Pharmaceutical Companies and Their Innovative Performance: The Mediating Role of Dynamic Capabilities and Corporate Social Responsibility

**DOI:** 10.5812/ijpr-135094

**Published:** 2023-06-19

**Authors:** Javad Shirmohammadi, Shahriar Azizi, Hamid Reza Rasekh, Farzad Peiravian, Mahyar Polroudi Moghaddam

**Affiliations:** 1 School of Pharmacy, Shahid Beheshti University of Medical Sciences, Tehran, Iran; 2 Department of Business Administration, Shahid Beheshti University, Tehran, Iran; 3 Department of Pharmacoeconomics and Pharma Management, School of Pharmacy, Shahid Beheshti University of Medical Sciences, Tehran, Iran

**Keywords:** Market Orientation, Dynamic Capabilities, Corporate Social Responsibility, Innovative Performance

## Abstract

**Background:**

Given the intensifying competition, adapting to the market environment and meeting customer demands are crucial aspects of the evolving marketing process. Market orientation (MO) represents an organizational culture encompassing shared beliefs and values that prioritize the customer's role in business planning.

**Objectives:**

This study seeks to explore the impact of MO on innovative performance (IP) and the potential mediating role of dynamic capabilities (DC) and corporate social responsibility (CSR) in this relationship.

**Methods:**

For this study, a structured quantitative questionnaire was distributed to 100 local pharmaceutical companies, resulting in 300 completed questionnaires. Each questionnaire consisted of four main components, which were filled out by three managers from each company: Chief executive officer (CEO), marketing manager, and research and development manager. The collected data were analyzed using SPSS software and structural equation methods to examine the research questions and hypotheses.

**Results:**

According to the study findings, there was a positive correlation between employee age, organizational structure, sales volume, and the presence of private companies with IP. MO, DC, and CSR showed a direct and significant relationship with IP. Moreover, the CSR of the company influenced IP through the mediating role of DC. Market orientation was found to enhance explorative IP, leading to improvements in existing processes and services.

**Conclusions:**

Based on the study results, it was found that MO has a direct positive impact on IP, leading to improvements in the company's existing processes through its influence on exploratory performance.

## 1. Background

A comprehensive understanding of marketing is essential for companies to accomplish their objectives. In today's fast-paced and ever-evolving landscape, marketing serves as a blend of art and science, playing a crucial role in the survival of manufacturing companies. The customer-centric approach has taken precedence over product-centric strategies, with marketing and market orientation (MO) assuming key roles. To sustain their position in the market, manufacturers must effectively engage and communicate with customers or buyers (1). As businesses face increasing complexity and fierce competition, marketers and company managers find themselves grappling with important questions. These include understanding customer needs and demands, identifying competitors, staying vigilant in the market, surpassing competitors in meeting customer needs, determining the most effective methods to achieve customer demands, and maximizing returns and performance from marketing capabilities and assets. To address these questions, an influence model of marketing knowledge management on business performance was developed, incorporating assets and dynamic capabilities (DC). In recent years, marketing knowledge management has emerged as a new concept that plays a crucial role in organizations. It involves harnessing marketing knowledge to fulfill customer needs, deliver superior value, gather and disseminate information from customers and competitors, and leverage it for strategic decision-making, planning, and interdepartmental coordination. Marketing knowledge management can enhance organizational learning, drive innovation, optimize organizational structure and strategies, and ultimately lead to a competitive advantage. To establish the concept of marketing knowledge management within organizations, it is essential to leverage operational concepts such as the capabilities of marketing knowledge management and market orientation (2). In recent times, there has been a global emphasis on corporate social responsibility (CSR) among companies, but Iranian firms have been relatively distant from adopting this perspective. While economic conditions may contribute to this slow progress in Iran, it can also be attributed to the attitudes and visions of senior managers within organizations. According to a theory proposed in recent years, business units have the potential to generate wealth, employment, and innovations while also strengthening their operations and fostering healthy competition. This can be achieved through collaborative efforts with society, creating suitable platforms for growth and advancement. Consequently, a company's responsibility toward society benefits not only the business unit itself but also the wider community. A deeper understanding of these advantages can lead to improved return on assets (ROA) for the firm (3).

Since this study focuses on the Iranian pharmaceutical industry, our initial analysis will explore the current status of the pharmaceutical market both at the national and global levels.

With recent developments in Iran's environment, the pharmaceutical market is expected to witness growth in both value and volume. Furthermore, the entry of private companies into the pharmaceutical market has contributed to its expansion. In an effort to enhance policy transparency, the Iran Food and Drug Administration (IFDA) has published its procedures in recent years. However, weaknesses such as lack of transparency, inflexibility, and inconsistency in adhering to regulations are still prevalent within the IFDA. The current management system within IFDA offices may result in different responses to similar requests from various pharmaceutical companies, often leading to decisions made on a case-by-case basis. Additionally, the lack of efficient control over promotional activities by pharmaceutical companies has contributed to increased demand in the Iranian market, potentially leading to excessive use of medication and increased financial burden on patients and IFDA members (4). A study on Iran's pharmaceutical exports revealed an unstable market structure, with Iraq, Afghanistan, and certain member countries of the commonwealth of independent states (CIS) being the primary importers of Iranian pharmaceutical products (5).

Market and customer orientation are essential characteristics of modern marketing, driven by the increasing level of competition. Customer retention is a critical task that requires unique techniques and tools. Marketers need to establish strong relationships with customers, provide appropriate services, and stay updated on competitors' activities. It involves not only retaining existing customers but also encouraging them to make additional purchases and ensuring their ongoing satisfaction. Achieving customer satisfaction requires the art and skill of inter-departmental coordination within the organization. Furthermore, with the globalization of trade and the integration of global markets facilitated by the World Trade Organization (WTO), which comprises full members representing about 97% of world trade and three-fourths of all countries, market orientation has become an inevitable consideration (6).

Organizations are compelled to deliver superior value to their customers by understanding their needs and serving them in the most effective way while also considering the external environment. In order to expand their operations, companies need to focus on other markets beyond the domestic market, as confining themselves solely to the domestic market is no longer sufficient. These markets offer higher quality but also carry higher risks. Additionally, the growth of e-commerce, increasing customer expectations regarding price and quality, advancements in information technology (IT) providing customers with greater knowledge, the development of networks and purchasing groups, a shift toward customer retention rather than just acquisition, and competition based on customer specifications and demands have all contributed to the growing importance of MO and DC (7).

In today's rapidly changing organizational environment, there is growing recognition among many organizations about the economic value of CSR. By integrating CSR into their core business practices, organizations can not only make a positive impact on society and the environment but also enhance their reputation and credibility. Stakeholders increasingly expect corporations to demonstrate social responsibility (SR) and are ready to acknowledge and criticize such behavior. Consequently, CSR offers mutual benefits, where the firm can gain from adopting an ethical and consistent approach while society and stakeholders develop a more favorable perception of the organization's performance and capabilities (8). The primary objective of this research is to examine the level of MO in companies and evaluate its impact on their innovative performance (IP). Furthermore, the study aims to prioritize different dimensions of MO based on firms' IP and investigate the mediating role of DC and CSR in the relationship between MO and IP. The findings of this study can provide valuable insights for researchers studying the management aspects of the Iranian pharmaceutical industry across different stages, including manufacturing, distribution, and sales.

## 2. Theoretical Foundation

### 2.1. Market Orientation

Market orientation is a strategic approach that prioritizes the creation and maintenance of superior customer value. It emphasizes the importance of employees in developing and leveraging market information (9).

Deshpandé and Farley (10) and Julian (11) defined MO as an organizational culture characterized by a shared set of values among employees regarding customers, emphasizing customers as the primary asset in business planning. Narver and Slater (9) and Dornberger et al. (12) also described MO as an organizational culture, but they further classified market-oriented firms as customer-oriented and competitor-oriented companies. According to Narver and Slater, competitor orientation is equally important to customer orientation for firms, highlighting the significance of inter-functional coordination (9, 12). Kohli and Jaworski conducted extensive field studies on MO and defined it as the driving force behind marketing within a company. They depicted MO as a collection of ideals pertaining to the generation of market intelligence, its dissemination across various organizational units, and the organization's responsiveness to it (13). When measuring MO, two commonly used definitions are MARKOR and MKTOR. The MKTOR definition consists of a 15-question, 7-point Likert scale with defined points. Within this definition, MO is comprised of three components: Customer orientation, competitor orientation, and inter-functional coordination. On the other hand, the MARKOR definition involves a 20-question, 5-point Likert scale assessing MO through three dimensions: Intelligence generation, intelligence dissemination, and responsiveness. MARKOR takes an organizational perspective of MO, while MKTOR focuses more on customer tendencies. We chose to adopt the MARKOR approach in our study, as it was focused on the organizational aspect (14).

Market intelligence typically refers to the awareness of external market factors (e.g., competition) (1) and the assessment of customer demands (1). However, these aspects alone do not fully capture the customer's perspective. Intelligence dissemination involves the necessary coordination among all departments within an organization to meet market needs. The third component of MO is responsiveness, which involves not only generating and disseminating knowledge but also actively addressing market demands. Responsiveness entails effectively responding to the knowledge produced and disseminated (13).

### 2.2. Dynamic Capabilities

Due to the rapid changes in the market environment and the growing need for organizational agility, companies are compelled to define DC as the ability to sense emerging opportunities, invest in capturing those opportunities, and reconfigure their resources and capabilities to adapt to changes (15). These three components of DC have been recognized as valuable sources and sustainable competitive advantages for organizations (16). The first component of DC is sensing, which refers to the organization's ability to quickly perceive market shifts and identify new possibilities. Continuously monitoring how environmental changes impact customer expectations is crucial for enhancing consumer acceptance and demand (17). Seizing is the second element of DC, enabling organizations to allocate the necessary investments to enact the required changes. This involves establishing appropriate protocols and possibly forming dedicated committees to oversee the development of new products. Configuration is the final aspect of DC, emphasizing a firm's capacity to implement new changes and generate new products while preserving existing processes within the organization (18).

### 2.3. Corporate Social Responsibility

The institute for CSR defines CSR as “the achievement of business success by upholding values and showing respect for people, society, and the environment” (19).

In his exploration of the holistic marketing concept, Kotler et al. suggests that this approach encompasses the awareness and acknowledgment of societal concerns, as well as the social, legal, environmental, and ethical dimensions of marketing plans and activities (20).

In our model, we incorporate a four-dimensional approach to CSR, which includes the following components:

(1) Economic component: The economic dimension of CSR is of utmost importance. Businesses serve as the primary economic entities in the community, producing goods and services that fulfill consumer needs and generating revenue through their sales.

(2) Legal component: The legal dimension enables businesses to demonstrate their commitment to societal well-being by adhering to relevant laws and regulations. Compliance with legal requirements is considered a fundamental aspect of SR.

(3) Ethical component: Ethical responsibilities encompass obligations that extend beyond legal requirements and are expected of community members. While there is a focus on ethical obligations, the definition of what is considered ethical can sometimes be subjective and unclear.

(4) Discretionary component: Discretionary responsibilities involve engaging in philanthropic activities that contribute to the betterment of society. Examples of this dimension include building homes for drug abusers and providing job training for the unemployed. The significance of these activities lies in the understanding that if a company chooses not to participate in them, it is not necessarily viewed as unethical (21).

### 2.4. Innovative Performance

This study assesses performance by considering the factor of IP, which is analyzed in terms of two dimensions: Explorative (EXPR) and exploitative (EXPA) IPs. The EXPA innovation scale focuses on the development of products, services, and processes that can enhance competition by leveraging and expanding the company's existing expertise. On the other hand, the EXPR IP pertains to the company's capacity to develop novel products and processes when its current expertise requires new knowledge and skills (22).

### 2.5. Hypotheses Development and Conceptual Model

**Figure 1.**
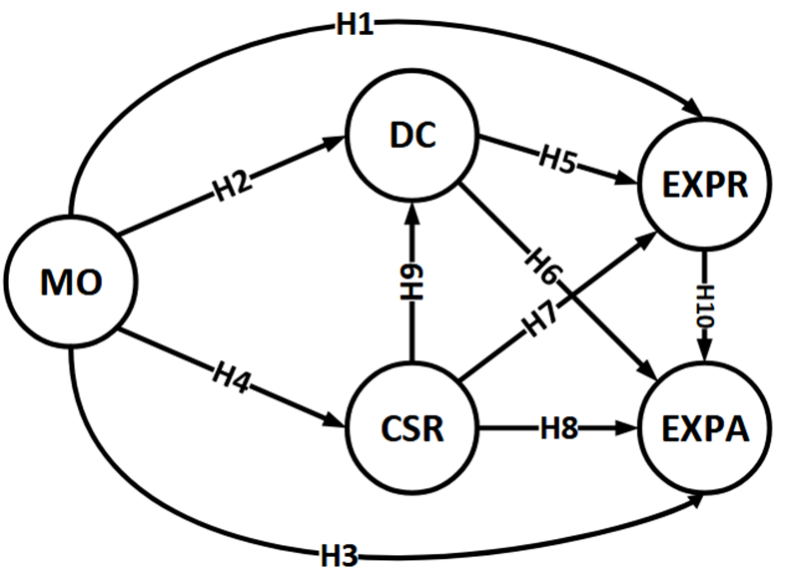
Hypotheses of conceptual model

A notable innovation of this study is examining the relationship between MO and IP, specifically in pharmaceutical companies, as there is currently no evidence available on this subject. To explore this relationship, we have modified several hypotheses. Figure 1 illustrates the hypotheses of our conceptual model.

(1) Market orientation has a direct positive impact on the company's IP, including both exploitative and explorative performance (H1, H3, H1-H10).

Numerous meta-analyses indicate that MO has the potential to enhance performance (23). The level of MO within an organization is likely to be influenced by its environmental context. In more competitive and dynamic contexts, organizations are expected to exhibit higher levels of market orientation. Consequently, the relationship between MO and performance is influenced by contextual factors specific to each organization. By enabling companies to understand their market environment, MO empowers them to adapt their products and services to meet customer demands effectively (24). Market orientation serves as a valuable tool for firms to identify customer needs and has been defined as a knowledge-based strategy for customer perception (25).

(2) Dynamic capabilities have a direct positive impact on the company's performance, including both exploitative and explorative performance (H5, H6, H5-H10).

In 2011, an article presented a structural model examining the relationship between DC and performance. The findings of the study suggested that dynamic capability and innovation are the primary factors influencing the performance of small and medium-sized firms (26).

In a study conducted in 2007, the results of the article indicate a strong and significant impact of dynamic capabilities on the innovation process.

A study conducted in 2017 establishes a significant relationship between DC with research and development (R&D) activities and IP (27).

(3) Corporate social responsibility has a direct positive impact on the company's IP, including both exploitative and explorative performance (H7, H8, H7-H10).

In a research study published in 2002, the financial performance of the banking industry in the Netherlands was compared with its CSR practices. The findings revealed a positive association between performance and CSR (28). Similarly, a study conducted in 2004 examined the relationship between CSR and the financial performance of companies, concluding that there is a positive effect between CSR and financial performance (19).

The research findings presented in the article indicated a weak and negative relationship between the financial performance and CSR of the company (29).

(4) Corporate social responsibility enhances company innovation through the mediating role of dynamic capabilities, including both exploitative and explorative performance (H9-H5, H9-H6).

Corporate social responsibility responsibilities foster collaboration among all stakeholders in both society and the company. In line with the innovation concept, prioritizing CSR cultivates trust between internal and external stakeholders, resulting in reduced costs associated with innovation in products and services. Considering that CSR and innovation typically operate in distinct departments within a business, it can be posited that DC acts as a mediator between the firm's IP and CSR. Dynamic capabilities facilitates the allocation of resources across different departments, enabling organizations to respond effectively to customer demands (30).

(5) Market orientation enhances company innovation through the mediating role of dynamic capabilities, including both exploitative and explorative performance (H2-H5, H2-H6).

Companies that prioritize MO exhibit greater adaptability to changes in the market environment (31). According to Day's research, MO fosters long-term capabilities in companies by enabling them to identify customer needs, competitor actions, and market trends. To cultivate these capabilities, executives must embrace market-oriented processes and behaviors within the organization (32). Naidoo (33) and Menguc and Auh (34) propose that adopting a MO approach facilitates the deployment of dynamic capabilities, ultimately contributing to firm growth. Fang et al. emphasize the significance of internal MO in enhancing organizational competencies. Hou has proposed that DC plays a vital role as a mediating factor, enhancing the performance impact of market orientation. In our study, we examine the influence of MO on a firm's IP, considering the mediating effect of DC. Given that MO is critical for the development of competencies in dynamic marketplaces, understanding this relationship is crucial (35, 36). Additionally, we included control variables, such as sales volume, ownership, firm age, and employee number, in the questionnaire to minimize potential confounding effects.

(6) Market orientation enhances company innovation through the mediating role of CSR, including both exploitative and explorative performance (H4-H7, H4-H8).

The use of a compatible market strategy that incorporates social, environmental, and economic responsibilities is crucial for managing firms toward sustainable market orientation. In today's consumer landscape, customers are more knowledgeable and demand a firm's dedication to CSR issues, necessitating new business strategies to create mutual value (37).

In a 2019 study on the mediating role of CSR between MO and IP, the article emphasizes the significance of linking CSR with a firm's MO efforts. It demonstrates how CSR enhances the firm's ability to address consumers' strategic demands (38).

In a 2012 study conducted in Malaysia, it was concluded that CSR plays a mediating role between MO and performance. This study highlights the strategic value of CSR in creating economic value for firms, even in an environment characterized by a concentrated ownership framework where firms may have a lower market focus (39).

## 3. Methods

At the onset of this study, in order to achieve the research objectives and validate the hypothesis, a questionnaire was administered to local Iranian pharmaceutical firms, and the obtained results were subsequently analyzed.

Convenience sampling was employed as the sampling methodology, as the companies under study were identified through the website of the Syndicate of the Owners of Human Pharmaceutical Industries of Iran. Visiting these companies in person, the study included those willing and able to participate by completing the questionnaire. The questionnaire, detailed in Table 1, encompassed four main categories. The questions within each dimension were derived from reputable studies. The first category consisted of the MO questionnaire (40), the second category focused on DC (41), the third category addressed corporate SR (42), and the fourth category explored IP (43).

**Table 1.** Category’s Dimensions of the Study

Dimension	Question No.
**1.1. MO-market intelligence generation**	7
**1.2. MO-market intelligence dissemination**	4
**1.3. MO-responsiveness to market intelligence**	6
**2.1. DC-sensing**	3
**2.2. DC-seizing**	4
**2.3. DC-configuration**	5
**3.1. CSR-economic citizenship**	4
**3.2. CSR-legal citizenship**	6
**3.3. CSR-ethical citizenship**	4
**3.4. CSR-discretionary citizenship**	6
**4.1. Innovative performance-exploratory innovation**	6
**4.2. Innovative performance-exploitative innovation**	5

Abbreviations: MO, market orientation; DC, dynamic capabilities; CSR, corporate social responsibility.

To ensure the validity of the questionnaire, two different approaches were employed. The first was face validity, which involved supervisors reviewing the questionnaire's appearance and items and providing feedback, which was incorporated into the questionnaire. The second approach was content validity, in which a group of subject-matter experts evaluated and assessed the questionnaire, making a few changes before approving it.

Regarding the reliability of the research tool, a pilot study was conducted with 30 experts in the research field. The results were analyzed using Cronbach's alpha method to measure internal consistency. The questionnaire demonstrated a Cronbach's alpha coefficient of 0.88, indicating a high level of internal correlation among the questionnaire items. This reliability test was further confirmed in Table 2, where the presence of thirty experts in the field supported the questionnaire's reliability.

**Table 2.** Reliability Test of the Questionnaire

Variable	Experts No.	Alpha
**Value**	30	0.88

The study population consisted of local Iranian pharmaceutical firms. A total of 387 questionnaires were distributed to the top four members of each company, including managing directors, R&D managers, marketing managers, and sales managers. The selection of companies was based on the Syndicate of the Owners of Human Pharmaceutical Industries of Iran, resulting in 129 companies being included in the study. Out of the distributed questionnaires, 300 were completed by representatives from 100 companies, yielding a response rate of approximately 80%.

The inclusion criteria for participation in the study involved companies being registered with the syndicate. To administer the questionnaire, face-to-face meetings were conducted with each member of the company, where the study questions were explained.

However, to mitigate information bias, which is a significant concern in completing the questionnaires, steps were taken to ensure that the quality of information remains consistent among comparison groups. To prevent evaluation bias, a subcategory of information bias, the questionnaire was divided based on the responder's specialty. Each participant only responded to the questions relevant to their area of expertise. Specifically, managers responsible for marketing, direction, and research and development were assigned separate sections of the questionnaire that addressed specific topics such as direct cost, SR, and IP. Subsequently, the validated questionnaire was distributed to experts from 100 Iranian local pharmaceutical companies. The collected data was analyzed using SPSS 21 and smart PLS 4 software, employing structural equation modeling.

The study process consisted of the following phases:

(1) Designing a questionnaire based on the research method.

(2) Validating the questionnaire.

(3) Collecting information from eligible individuals, including criteria members, through the questionnaire.

(4) Evaluating and analyzing the questionnaire results.

(5) Discussing, concluding, and testing the hypotheses.

Additionally, we conducted a survey involving 30 pharmaceutical experts who possess experience in the pharmaceutical market and have knowledge of launching new medicines into the market.

### 3.1. Data Analysis

Bartlett's test of sphericity was utilized to examine the hypothesis that “the correlation matrix of observed variables is an identity matrix (44).” By evaluating the significance level of the chi-squared test, this assessment confirms the absence of correlations among the variables. If the P-value of Bartlett's test is less than 5%, it indicates that the matrix is not identified, suggesting a correlation between the variables and rejection of the null hypothesis. In Table 3, the test's significance level is presented as 0.001, supporting the rejection of the null hypothesis and indicating a significant correlation between the variables. Hence, with a statistical confidence of 95%, the parametric statistical test can be employed, and the sample size is deemed sufficient for conducting factor analysis based on the Kaiser-Meyer-Olkin (KMO) test result (45).

**Table 3.** Kaiser-Meyer-Olkin and Bartlett's Test for Sampling Adequacy

	Values
**Kaiser-Meyer-Olkin measure of sampling adequacy**	0.882
**Bartlett's test of sphericity**	
Approx. chi-square	7.542E3
Degrees of freedom	1689
Significance level	0.001

Based on the KMO test presented in Table 1, the number of questionnaires appears to be sufficient, and Cronbach's alpha value for each dimension of the questionnaire exceeds 0.7.

Due to the good validity of the questionnaires in testing the hypotheses, we utilized smart PLS software and path analysis for this study. Path analysis is employed to evaluate a system of equations that includes all variables. Path models can encompass multiple dependent variables. In SmartPLS, a path model's parameter can consist of single-parameter constructs. When constructing a score for a variable based on multiple indicators, all indicators are assigned equal weight. In essence, the model focuses on the structural significance of the relationships between the observed variables and the control variables. This type of model is commonly used when one or more parameters mediate the relationship between two other variables (mediation models). Additionally, moderated mediation can also be modeled.

## 4. Results and Discussion

**Figure 2.**
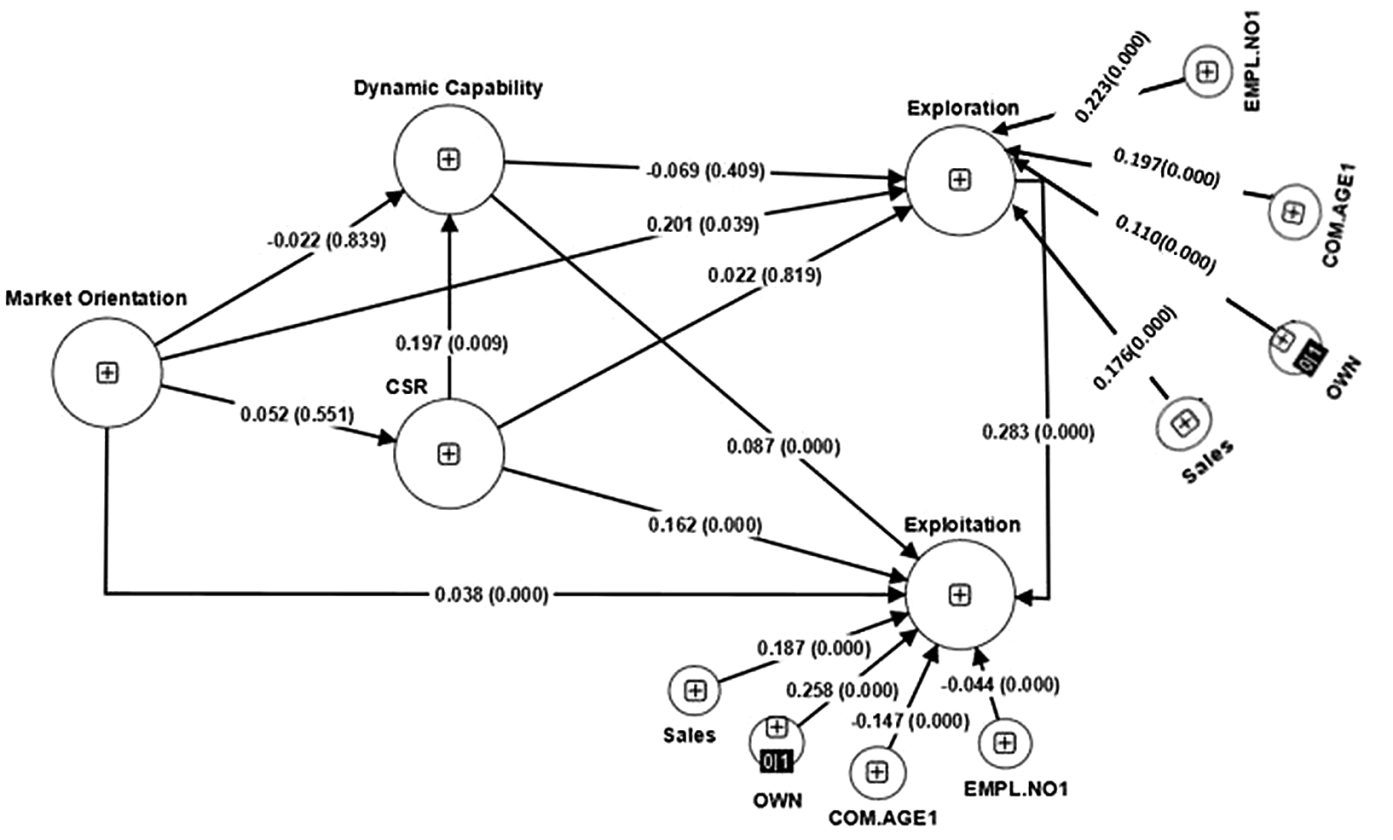
The results of the model hypotheses test. Sales: Sales of each company; OWN: Ownership of company (private or governmental); COM.AGE1: Company age; EMPL.NO1: Employee number of each company.

Figure 2 illustrates the testing of hypotheses and the inclusion of control variables in the context of innovation. Initially, we examine the impact of control variables and subsequently analyze the hypotheses. It is important to note that due to the practical nature of control variables and their dimensions, we demonstrate that relying on management's budget allocation authority can influence MO, DC, and CSR policies. Therefore, we assess the influence of control variables on IP.

### 4.1. Demographic Characteristics

According to the findings presented in Figure 2, the age range of 11 to 20 years has the highest frequency among the studied companies, accounting for 28% of the sample. It is noteworthy that younger companies within this age range tend to exhibit higher innovative capabilities. Existing research suggests that as companies age, their engagement in innovative activities tends to decrease (46, 47).

Based on the publications and experience of WTO (WTO deals with the global rules of trade between nations. Its main function is to ensure that trade flows as smoothly, predictably, and freely as possible.) members, the successful development of a new product typically takes 10 to 15 years. Additionally, the chance of obtaining approval for a drug to enter phase 1 clinical trials ranges from 7% to 45%. However, it should be noted that Iran, not being a WTO member, experiences a shorter timeline for Iranian companies, estimated to be around 3 to 5 years based on expert opinions.

Out of the 466 new innovative materials imported to the American market between 1991 and 2009, only 10% of them achieved sales exceeding $10 billion during their patent life cycle (48).

Based on the research findings regarding the number of workers, 27 firms had between 101 and 200 employees, indicating that some study participants had increased agility due to a smaller workforce and fewer divisions. According to the statistics from 2020 to 2021 on the ranking of companies based on their number of employees, Pfizer Inc. claimed the first rank in innovation with $81.3 billion in revenue and 79,000 employees. AstraZeneca PLC secured the second rank with $37.4 billion in revenue and 83,100 employees, while Regeneron Inc. attained the third rank with $16.1 billion in revenue and 10,368 employees. Johnson & Johnson Inc. held the fourth rank with $93.8 billion in revenue and 141,700 employees, and Merck & Co. Inc. obtained the fifth rank with $48.7 billion in revenue and 67,500 employees (49).

There is a direct and negative relationship (0.044) between the number of employees and innovation. To investigate this, two of the largest and most profitable companies globally were examined. The findings revealed that a significant portion of the drugs sold by these companies were not developed in-house. Out of a total of 62 products (44 from Pfizer and 18 from J&J), only 10 of Pfizer's 44 products and two of J&J's 18 products were developed in-house. According to the article, approximately 81% of new products in these companies are manufactured by intermediary companies (50).

According to the model test, the age of the company as a control variable has a negative and direct effect on performance, indicated by a coefficient of -0.147. This implies that as the company's age increases, innovation is likely to decline. This finding is consistent with numerous studies that suggest older firms have lower probabilities of innovation (51-53).

Based on the results of the model test, the company's sales amount has a direct and significant relationship (0.187) with IP. This means that as the company's sales increase, so does its IP. This finding is consistent with previous studies that have also shown a positive correlation between sales amount and IP (54, 55).

According to the model test, private companies have exhibited a higher level of innovation (0.258) compared to public companies. This finding is consistent with numerous studies that have reported similar results (56, 57).

Based on Table 4 and Figure 2, the analysis of hypotheses and the impact of control variables on IP have been conducted. It has been observed that company age and employment number have a direct, significant, and negative effect on IP, as discussed in the demographic characteristics section.

Sales volume and private ownership have a positive and significant influence on a company's innovation performance. This indicates that higher sales volume is associated with greater innovation in processes. The hypothesis that MO improves IP and enhances exploitative performance through the mediating role of explorative performance was supported. However, the mediating role of DC and CSR between MO and IP was not supported and rejected.

The hypothesis that DC enhances exploitative performance was supported, while the relationship between explorative performance and DC was not supported and rejected.

The hypothesis that CSR enhances exploitative performance was supported, while the relationship between explorative performance and CSR was not supported and rejected. Additionally, the mediating role of DC on exploitative performance and CSR was confirmed. The next section describes these hypotheses.

**Table 4.** The P-Value and Coefficient of Each Relationship ^a^

	Original Sample (O)	T Statistics (|O/STDEV|)	P-Values
**COM.AGE1 → EXPA**	-0.147	0	0
**COM.AGE1 → EXPR**	0.197	0	0
**CSR → EXPR**	0.022	0.229	0.819
**CSR → EXPA**	0.162	0	0
**Dynamic capability → CSR**	0.196	2.58	0.01
**Dynamic capability → EXPR**	-0.069	0.826	0.409
**Dynamic capability → EXPA**	0.087	0	0
**EMPL.NO1 → EXPA**	-0.044	0	0
**EMPL.NO1 → EXPR**	0.223	0	0
**EXPR → EXPA**	0.283	0	0
**MO → CSR**	0.052	0.674	0.5
**MO → Dynamic capability**	-0.022	0.917	0.839
**MO → EXPR**	0.201	2.062	0.039
**MO → EXPA**	0.038	0	0
**OWN → EXPA**	0.258	0	0
**OWN → EXPR**	0.110	0	0
**Sales → EXPA**	0.187	0	0
**Sales → EXPR**	0.176	0	0

^a^ COM.AGE1: Company age; CSR: Corporate social responsibility; EXPR: Explorative; EXPA: Exploitative; EMPL.NO1: Employee number of each company; MO: Market orientation; OWN: Ownership of company (private or governmental); Sales: Sales of each company.

### 4.2. Market Orientation and Innovative Performance

Organizational performance encompasses various dimensions. A holistic perspective of organizational performance includes not only financial indicators but also other measures that evaluate value generation within an organization (58).

This study demonstrates a significant relationship between MO and IP in Iranian pharmaceutical companies. Numerous studies have substantiated the direct impact of MO on IP across diverse industries.

A Turkish study conducted in 2011 on companies from various industries, including food, chemical, and ironwork, among others (excluding the pharmaceutical industry), reveals a direct and significant relationship between MO and IP (59).

According to a 2017 research conducted in Albania on 99 different industrial enterprises, the relationship between MO and IP is significant and noteworthy. However, it is important to note that the conclusions are most relevant when considering sector-specific firms, as each industry may yield specific findings. For instance, in industries with lower or slower innovation development, MO may not exhibit a positive and significant relationship with IP (60).

A Colombian study conducted in 2018 on innovative national companies indicated that the industry's environment directly influences the relationship between MO and IP (61).

### 4.3. Dynamic Capabilities and Innovative Performance

As mentioned in the findings section, there is a direct and significant relationship between DC and IP, which aligns with the results of previous studies. For instance, a Nigerian study titled “absorptive capacity, DC, and innovation commercialization in Nigeria” conducted in 2019 also demonstrated a direct and significant relationship between DC and IP, highlighting DC as a key determinant of how new products are marketed (62).

A study conducted in 2022 titled “hegemony of digital platforms, innovation culture, and e-commerce marketing capabilities: The innovative performance perspective” concluded that digital platforms mediate the relationship between e-commerce DC and IP (63).

A Chinese study conducted in 2011 demonstrated a significant relationship between DC and IP, with knowledge integration capability acting as a mediator (64).

### 4.4. Corporate Social Responsibility and Innovative Performance

The results indicate a strong and significant connection between the success of pharmaceutical companies in innovation and CSR. When CSR was evaluated across its economic, legal, ethical, and philanthropic dimensions, exploratory innovation performance was found to be significantly associated with CSR. A study examining the correlation between CSR and financial performance also revealed a direct and significant relationship between financial performance and CSR (65).

In a 2019 study examining the impact of CSR on service innovative performance through the lens of DC, it was concluded that environmental and SR, with the mediating effect of DC, contribute to enhancing IP (66).

In a 2019 study, a significant and positive relationship was observed between CSR and organizational performance, with knowledge management serving as a mediating factor (67).

### 4.5. The Mechanism of Market Orientation in Innovative Performance

The existing research literature indicates that the three components discussed in this study, namely MO, CSR, and DC, can have an impact on IP.

A. Market orientation and dynamic capabilities: The findings indicate that in Iranian pharmaceutical companies, MO does not have the ability to improve IP through the mechanism of DC. One possible reason for this could be the slow R&D process in Iran and the lack of focus on innovation and R&D in pharmaceutical companies. On the other hand, DC is associated with rapid changes in the pharmaceutical environment, the long-term utilization of senior managers to achieve strategic goals, and a lack of attention to future opportunities, such as exporting for pharmaceutical companies in the Iranian market. It is worth noting that this relationship is more commonly studied in terms of financial performance, and the relationship between these two variables is less explored in the context of IP.

A study conducted in 2008 demonstrated that MO has a positive impact on financial performance with the mediating effect of DC (36).

In a study conducted in 2016 titled “MO, marketing capability, and new product performance: The moderating role of absorptive capacity,” it was concluded that focusing on MO and DC can lead to a competitive advantage in business performance, and MO can enhance the business performance of new products through DC (68).

A study carried out in 2015 titled “the mediating role of innovation capability on MO and export performance” revealed that innovation capability acts as a mediator between MO and innovation as well as export performance. The findings of this study suggest that both innovation capabilities and MO can contribute to gaining a competitive advantage and increasing export performance (69).

B. Market orientation and corporate social responsibility: The research findings suggest that MO does not improve IP through the mediating role of CSR. This result contradicts the findings of most previous studies, which have highlighted the lack of coordination among different divisions within a company as a possible underlying factor. Additionally, the study found that the SR component of the company positively influenced innovation performance through the mediating effect of DC.

In a study conducted by Grinstein in 2010 titled “the effect of market orientation and its components on innovation consequences: A meta-analysis,” it was concluded that there is no mediating relationship between MO and CSR on IP (70).

In a study conducted in 2013 titled “MO and learning ability and CSR on IP,” it was concluded that CSR does not act as a mediating link between learning and MO in relation to innovative performance (71).

In a study conducted in 2012 titled “MO, innovation, and CSR in technological companies in Ghana,” it was concluded that MO and CSR have a direct relationship with innovation. Additionally, CSR serves as a mediator between MO and IP (72).

### 4.6. Research Limitations

(1) The current study employed a questionnaire as the survey instrument, potentially leading to individuals declining to provide honest responses and offering inaccurate answers.

(2) This study is cross-sectional in nature, making it challenging to establish causality.

(3) The extensive length of the questionnaire and the subsequent time required to complete it may have influenced the response accuracy of participants.

(4) This study assessed multiple parameters, some of which could be influenced by external factors, and a large number of these parameters were not implemented during the study's execution phase.

### 4.7. Conclusions

Based on the obtained results, the answer to the main hypothesis is that the mechanism of the effect of MO on innovative performance is the direct effect of MO on the IP of pharmaceutical companies in Iran.

According to the results of the findings, MO via the mechanism of DC does not improve IP in Iran's manufacturing pharmaceutical companies, which can be attributed to the slowness of the research and development process in Iran, less attention to research and development, and innovation processes in Iran's pharmaceutical companies. This is compounded by the lack of stability and long-term use of upstream managers to achieve the company's strategic goals, as well as the insufficient attention given to opportunities such as exports in the Iranian market.

Hence, this relationship has primarily been studied in the context of financial performance, with limited research on its connection to IP. Furthermore, the focus of these investigations has primarily been on manufacturing pharmaceutical companies, further highlighting the need for more studies in this area.

Dynamic capabilities exhibits a direct and significant correlation with IP, consistent with findings from previous studies that align with the current research. Notably, profitable innovation showcases a direct association. Additionally, the company's SR demonstrates a direct and significant link with IP, which can be attributed to the attention given to this aspect by younger companies. It is observed that IP activities are predominantly carried out in younger companies due to their heightened agility.

In addition to its direct impact on profit-oriented IP, MO also influences utility-based IP through the mediating role of innovative exploratory performance. This suggests that MO has enhanced processes and services that were previously lacking in terms of this aspect.

## Data Availability

The data are not publicly available due to the first publication of this article in this journal.
